# Severe thrombocytopenia in primary EBV- Infection with no signs of infectious mononucleosis. A case report

**DOI:** 10.1016/j.idcr.2022.e01643

**Published:** 2022-11-01

**Authors:** Maria Pishmisheva-Peleva, Stanislav Kotsev, Djahide Emin, Naum Simonoski, Martina Shopova, Radka Argirova

**Affiliations:** a Department of Infectious Diseases, Pazardzhik Multiprofile Hospital for Active Treatment, Pazardzhik, Bulgaria; b Second Department of Internal Diseases – Haematology Ward, Pazardzhik Multiprofile Hospital for Active Treatment, Pazardzhik, Bulgaria; c National Specialised Hospital for Active Treatment of Haematological Diseases, Sofia, Bulgaria; d Laboratory of Virology, Acibadem City Clinic, Tokuda Hospital, Sofia, Bulgaria

**Keywords:** Thrombocytopenia, EBV-infection, Infectious mononucleosis, EBV DNA, Haemorrhagic diathesis

## Abstract

Epstein-Barr virus is a widely spread Herpesvirus. Primary EBV infection affects children and young people, inducing haematological changes, with lymphocytosis being the most common. Moderate symptomless thrombocytopenia is found in 50% of the patients, however, severe thrombocytopenia is exceptionally rare. We present a case report of a 20-year-old man with an acute EBV infection, severe thrombocytopenia, and no signs of infectious mononucleosis.

## Highlights

• EBV infection usually causes infectious mononucleosis in children and young adults.
• EBV induces haematological changes, but severe thrombocytopenia is rarely observed.
• We observed severe thrombocytopenia in EBV infection without mononucleosis signs.
• This case report presents the diagnostic and therapeutic challenges we faced.

## Introduction

Epstein-Barr virus (EBV) is a widely spread oncogenic virus. About 90–95 % of the population have serological evidence of previous infection [6]. Immunocompetent children and young people typically experience an asymptomatic infection. Primary EBV infection generally presents as infectious mononucleosis (IM). After contagion the initial viral replication is suppressed thus establishes a lifelong latent infection [10], associated with nasopharyngeal carcinoma, Burkitt lymphoma, Hodgkin disease and EBV-related lymphoproliferative disorders. EBV (Human herpesvirus 4) was found by electron microscopy of a Burkitt lymphoma-derived cell line from a child. As a herpesvirus, EBV possesses DNA, nucleocapsid and viral envelope [2]. EBV targets the nasal and oropharyngeal epithelium, and B-lymphocytes, binding to CD21. In contrast to the in vitro lysis of the infected epithelium, in B-lymphocytes EBV remains latent and activates spontaneously in a little percent. The viral capsule antigen – VCA, early antigen – EA and nuclear antigen – EBNA are of greatest diagnostic significance. VCA and EA appear with the symptom’s onset, while EBNA – 2 – 3 months later.

IM presents with fever, tonsillopharyngitis and lymphadenopathy, accompanied by malaise, hyponasal speech, swollen face, and distinctive blood count abnormalities. A few of the patients have hepatosplenomegaly or splenomegaly. IM has a benign course and resolves in three weeks, but malaise might persist for months. Diagnosis is usually based on serological tests. The profile of an acute infection includes EBV VCA IgM and IgG (+) pos., EBNA (-) neg. Despite the presence of specific antibodies, EBV might persist in the organism some months after infection. Hepatitis, obstructive respiratory failure, neurological disorders, interstitial nephritis, myocarditis, and spleen rupture are infrequent complications. Neutropenia with atypical lymphocytosis is seen in 50 – 80% of the cases, haemolytic anaemia – in 3%, and moderate thrombocytopenia in 25 – 50%. Severe thrombocytopenia (<20 G/l) is extremely rare [8] – a thorough literature search has shown few publications. In view of this, we present a case report of an acute EBV infection with severe thrombocytopenia.

## Case report and results

A 20-year-old patient presented to hospital with one-day history of fever, malaise, rash, nasal and gingival bleeding. He had spent the previous 3 days in the mountain. He denied thick bites, consumption of water or food approached by rodents and contact with ill people. The patient had no comorbidities; he had not had COVID-19 or vaccination against it.On admission, the patient was conscious and alert with subfebrile temperature – 37,7 °C. Variously shaped haemorrhages covered the gingiva, buccal mucosa, and lips; the soft palate and tonsils were intact. Non-itchy maculopapular rash and petechiae (d = 0.5 mm) spread on the trunk and limbs. The abdominal palpation revealed hepato/splenomegaly – 1/3 cm below the costal margins. No signs of craniopharyngeal syndrome, lymphonodulomegaly, respiratory, cardiovascular, neurological, and renal disorders presented.

The laboratory tests revealed normal leucocytes and erythrocytes, but extreme thrombocytopenia – 5G/l. (Remark: 5G/l is the lowest platelets count detectable by the laboratory equipment. Even lower, the platelets count would be given 5 G/l). The biochemistry laboratory tests were within normal ranges. Regarding the vasculitis, an immunological assay was also performed – ANA-screening test was negative. The blood smear interpretation showed: bands – 2 %, neutrophils – 29 %, lymphocytes – 63 %, and monocytes – 6 %. The lymphocytes were mature, but different in size – some of them were small, whereas the others were bigger and with abundant cytoplasm. Lympho-monocytes were also detected. The erythrocytes were with normal morphology. Atypical cells and platelets were not found.

The diagnosis was based on the serological tests – the serum sample was positive for anti-EBV-CA IgM and EA-D, but negative for EBNA. Along with that, extremely high concentration of EBV DNA was detected in a saliva sample. Based on these results, the patient was diagnosed with an acute EBV infection. The diagnostic test results are shown in Table 1.

**Table1:** Diagnostic tests results.

Diagnostic test		Result
anti-HAV IgM HBsAg anti-HBc anti-HCV anti-HIV anti-SARS-CoV-2 IgM/IgG		Negative
anti-HBs		Positive
**CMV IgM**		**Weakly positive**
CMV IgG		Negative
EBV immunoblot	**EBV-CA IgM**	**(+)**
	**VCA gp125**	**(++)**
	**VCA p19**	**(+++)**
	EBNA –1	Negative
	**EA–D**	**(+++)**
ЕBV avidity		20% - recent infection
PCR EBV DNA quantitative analysis*		770,664.82 IU/ml

* Remark: PCR assay of a buccal swab.

The abdominal ultrasonography revealed only splenomegaly (d = 77,4 mm). The treatment included intravenous solutions, vessel strengthening and haemostatic medications, vitamins, and antibiotic (Cefoperazone 2 g/24 h). Corticosteroid (Methylprednisolon 120 mg/24 h in 3 equal doses) was initiated immediately after the blood collection. Seven units of platelet concentrate were transfused in 3 different days. Despite patient’s stable condition, on the 2nd day macroscopic haematuria and new vasculitic lesions on the limbs occurred.By the 10th day the thrombocytes increased to 12 G/l and the haemorrhagic syndrome was controlled. The platelets reached 50 G/l by the 15th day. Methylprednisolone was given for 30 days, with dose reduction after the 15th.

On discharge, the patient was clinically well, with normal laboratory values and normal size of the spleen on control ultrasonography. A month after the disease onset a control PCR test detected 3 118,045,24 IU/ml EBV DNA. Despite the high viral load, treatment was not initiated, as there were no signs and symptoms of a disease.

Fever, lymphadenopathy and tonsilitis, typical of IM were missing during the hospital stay and the outpatient observation.

## Discussion

The mechanisms of virus-induced thrombocytopenia are still unclear. It might be a consequence of 1. Aggregation – platelets agglutination or leucocytes adhesion typically occur in systemic and viral diseases, therefore distinguishing thrombocytopenia from pseudothrombocytopenia is essential. 2. Impaired thrombopoiesis – direct viral invasion of haemopoietic stem cells leads to imperfect thrombopoiesis and consequent thrombocytopenia. The lower cytokines and hepatic thrombopoietin levels in the infected organism probably play a role, as well [4]. 3. Increased platelet destruction – being a commonplace of advanced infection, the increased platelet destruction induces rapid thrombocytopenia by direct virus – thrombocyte interaction or by the mediation of toll-like receptors and integrins, that trigger platelets activation, degranulation, hepatic, and splenic clearance [4], [9]. 5. Platelets function as antigen presenting cells (APCs). Evidence has shown that thrombocytes and megakaryocytes possess the necessary MHC-I and co-molecules to function as APCs [9]. 5. Presence of antiplatelet antibodies. The EBV-infected B-cells produce heterophile antibodies that might autoreact with platelets and erythrocytes, leading to thrombocytopenia and haemolytic anaemia [3]. Overall, thrombocytopenia in viral infections is presumably a multifactorial disorder. Corticosteroids (Dexamethasone, Methylprednisolon, Prednisone) are first line medications in treating thrombocytopenia. Steroids decrease platelets destruction, increase thrombopoiesis; reduce antiplatelet antibody concentration, dissolve platelet – antibody complex; strengthen vessels and reduce bleeding. Their onset of action is between the 2nd – 14th day after initiation reaching peak effect between the 4th – 28th day. Acyclovir administration in EBV infection [5] is still controversial. Despite the viral load reduction, significantly alteration of the clinical course was not observed. Venous immunovenin rapidly increases the platelets count with onset of action between the 1st – 3rd day after initiation, reaching peak effect on the 3rd – 7th day. Anti-D- immunoglobulin is preferred in Rh (+) positive patients with intact spleen. It has no effect in Rh (-) negative and post splenectomy, moreover, it might trigger haemolytic anaemia. Second line treatment include monoclonal antibodies – Retuximab; immunosuppressive agents, platelets antagonists, and splenectomy [1]. Platelet concentrate should be transfused after an antiplatelet antibody test, splenectomy or before surgery.

This case is peculiar, because: (1) there were no typical manifestations of IM; (2) it was the first time we had observed a vasculitis that resolved with the haemorrhages improvement; (3) it was the first time we had diagnosed such severe thrombocytopenia.

Antiplatelets antibodies test was not performed because of the early steroid initiation. Owing to that, having performed the test, the result would have been unreliable.

Although severe thrombocytopenia is an extremely rare complication of an acute EBV infection [7], it is a potentially life-threatening condition. Establishing an aetiological diagnosis might be difficult if the cardinal symptoms of a disease are missing. Such conditions are challenging and acquire a broad spectrum of knowledge, wide range of diagnostic tests, a proper strategy, as well as patience, and patient’s reliance.

## Ethical approval

A written informed consent was obtained from the patient.

## Consent

Written informed consent was obtained from the patient for publication of this case report and accompanying images. A copy of the written consent is available for review by the Editor-in-Chief of this journalon request.

## **Author contribution**

MP, SK and MS were involved in the diagnosis establishment and treatment of the patient. DE and NS performed the haematological consultation. RA performed the PCR tests. All authors read and approved the final manuscript.

## Funding

No funding was provided for this case report.

## Conflict of interest

The authors declare no conflict of interests.
